# Maltine and Its Combinations

**Published:** 1879-02

**Authors:** 


### Maltine and its Combinations.

Our experience with this comparatively new Medicine has been confined to cases of derangement of digestion and imperfect nutrition, and in all instances thus for patients have expressed great satisfaction in its use, in some instances attributing to it most wonderful effects. It is growing in popular favor and is worthy of trial.

